# Supplementary material for: A systematic assessment of preclinical multilaboratory studies and a comparison to single laboratory studies
Source: eLife. 2023 Mar 9;12:e76300. doi: 10.7554/eLife.76300 (PMC10168693; doi:10.7554/eLife.76300)

Figure 2 – figure supplement 1. Standardized mean differences for all 14 single vs multilaboratory comparisons and sub-groups by total quality score

Study name	Time point	Single vs Multi	Std diff in means	Lower limit	Upper limit
Reimer	1985	Multi	-0.08	-0.70	0.53
**Multi**			-0.08	-0.70	0.53
Jugdut (a)	1980	Single	-1.41	-2.26	-0.55
Romson (a)	1980	Single	-2.65	-4.35	-0.95
Jugdut (b)	1981	Single	-1.15	-1.84	-0.45
Darsee	1981	Single	-1.70	-2.85	-0.55
Romson (b)	1982	Single	-1.24	-2.13	-0.35
Kirlin	1982	Single	-0.43	-1.46	0.59
Brown	1983	Single	0.92	0.01	1.82
Flynn	1984	Single	-1.00	-1.68	-0.33
Jugdut (c)	1985	Single	-0.79	-1.47	-0.12
Allan	1985	Single	1.28	0.23	2.32
**Single**			-0.76	-1.36	-0.16

(Statistics for each study: Std diff in means, Lower limit, Upper limit)

Std diff in means and 95% CI

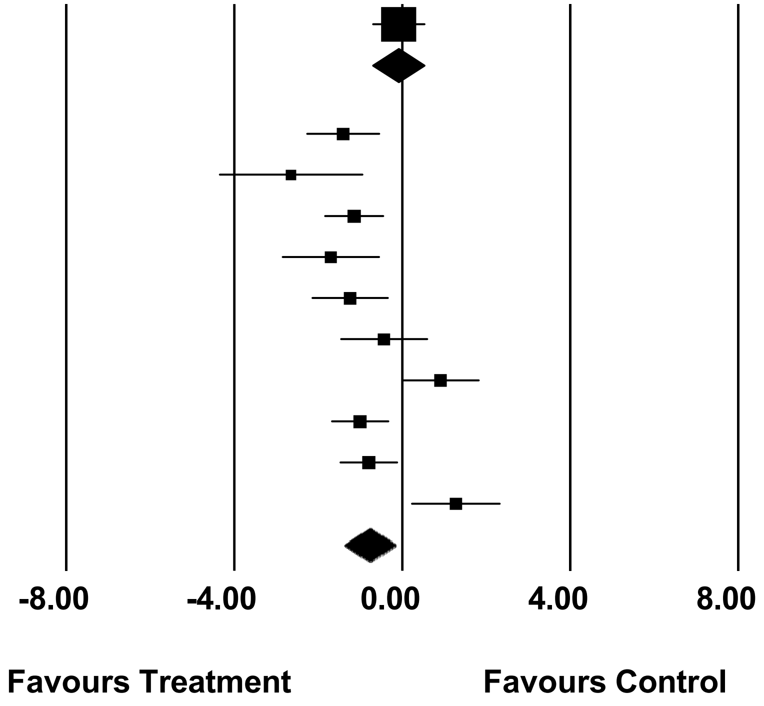

Study name	Time point	Single vs Multi	Quality Score	Statistics for each study: Std diff in means	Lower limit	Upper limit
Reimer	1985	Multi	<3	-0.08	-0.70	0.53
**Multi OVERALL**				-0.08	-0.70	0.53
Jugdut (a)	1980	Single	<3	-1.41	-2.26	-0.55
Romson (a)	1980	Single	<3	-2.65	-4.35	-0.95
Jugdut (b)	1981	Single	<3	-1.15	-1.84	-0.45
Darsee	1981	Single	<3	-1.70	-2.85	-0.55
Romson (b)	1982	Single	<3	-1.24	-2.13	-0.35
Kirlin	1982	Single	<3	-0.43	-1.46	0.59
Brown	1983	Single	<3	0.92	0.01	1.82
Flynn	1984	Single	<3	-1.00	-1.68	-0.33
Jugdut (c)	1985	Single	<3	-0.79	-1.47	-0.12
Allan	1985	Single	<3	1.28	0.23	2.32
**Single OVERALL**				-0.76	-1.36	-0.16

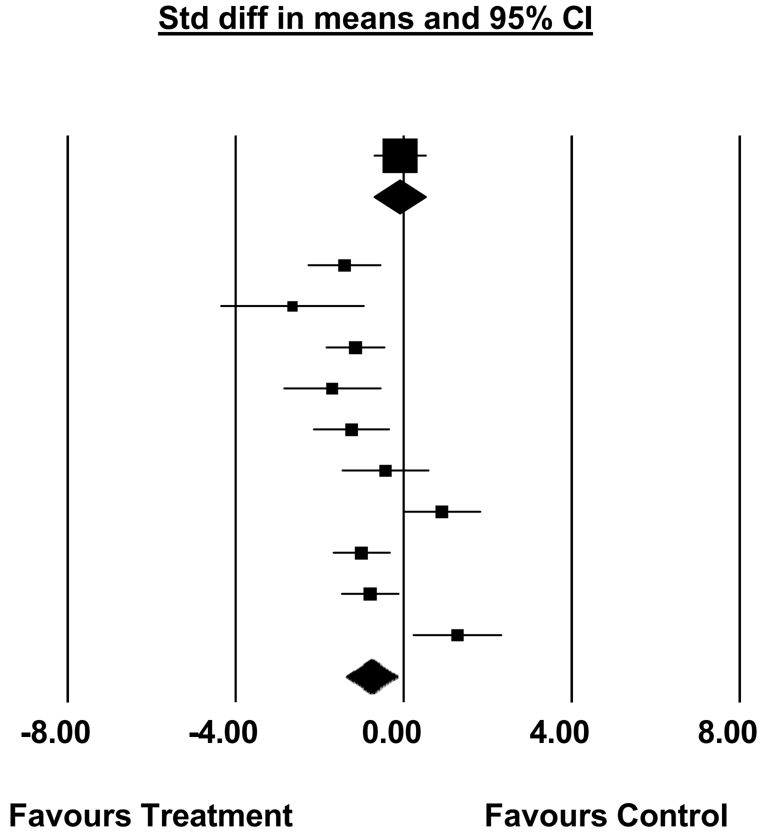

Study name	Time point	Single vs Multi	Std diff in means	Lower limit	Upper limit
Alam	2009	Multi	2.19	1.25	3.12
**Multi**			**2.19**	**1.25**	**3.12**
Alam	2011	Single	6.75	4.13	9.37
Lee	2013	Single	0.58	-0.32	1.47
Schechtman	2019	Single	0.53	-0.10	1.16
Sheppard	2018	Single	1.00	-0.20	2.20
Sheppard	2019	Single	0.69	-0.14	1.51
**Single**			**1.28**	**0.25**	**2.31**

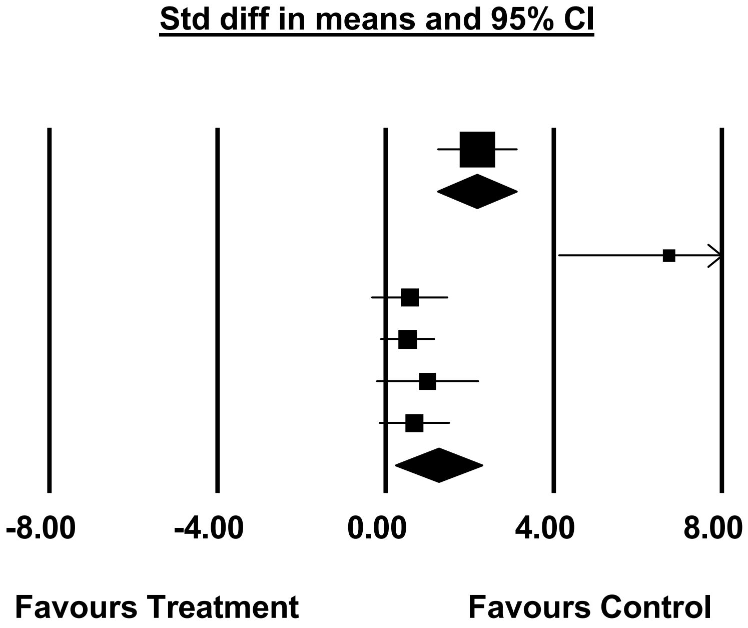

Study name	Time point	Single vs Multi	Time point	Std diff in means	Lower limit	Upper limit
Alam	2009	Multi	<3	2.19	1.25	3.12
**Multi OVERALL**				**2.19**	**1.25**	**3.12**
Alam	2011	Single	<3	6.75	4.13	9.37
Lee	2013	Single	<3	0.58	-0.32	1.47
Schechtman	2019	Single	<3	0.53	-0.10	1.16
Sheppard	2018	Single	<3	1.00	-0.20	2.20
Sheppard	2019	Single	<3	0.69	-0.14	1.51
**Single OVERALL**				**1.28**	**0.25**	**2.31**

(Statistics for each study)

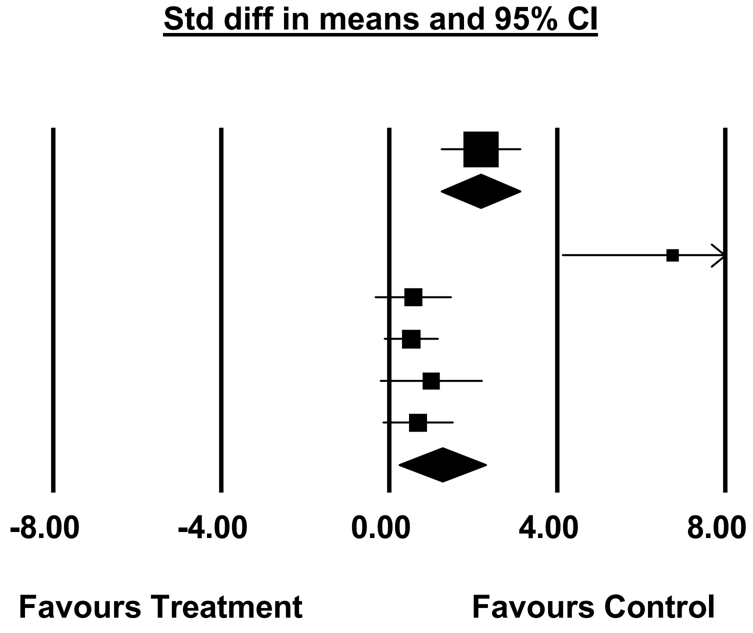

Study name	Time point	Single vs Multi	Std diff in means	Lower limit	Upper limit
			**Statistics for each study**		
Spoerke	2009	Multi	0.56	-0.44	1.56
**Multi**			**0.56**	**-0.44**	**1.56**
Shuja	2008	Single	-0.27	-1.41	0.87
Shuja	2011	Single	-0.65	-1.93	0.62
Imam	2013	Single	0.52	-0.74	1.78
Halaweish	2016	Single	-1.31	-2.67	0.06
Georgoff	2017	Single	-0.08	-1.32	1.16
**Single**			**-0.32**	**-0.89**	**0.24**

Std diff in means and 95% CI

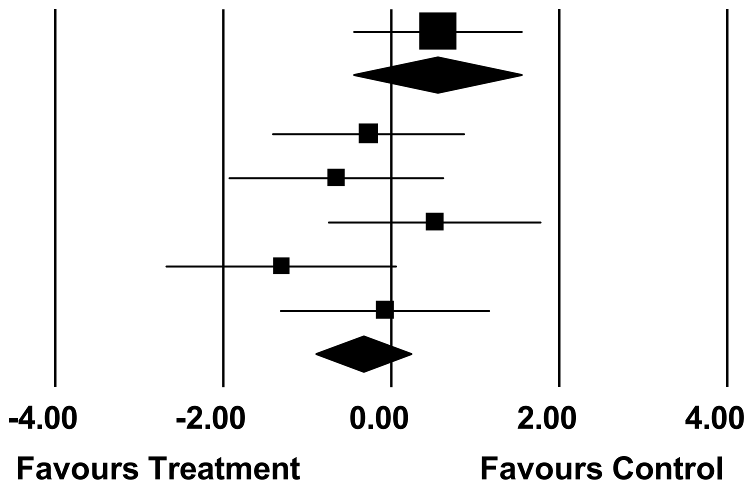

Study name	Time point	Single vs Multi	Quality Score	Statistics for each study			Std diff in means and 95% CI
				Std diff in means	Lower limit	Upper limit	
Spoerke	2009	Multi	<3	0.56	-0.44	1.56	
**Multi OVERALL**				**0.56**	**-0.44**	**1.56**	
Shuja	2008	Single	<3	-0.27	-1.41	0.87	
Shuja	2011	Single	<3	-0.65	-1.93	0.62	
Imam	2013	Single	<3	0.52	-0.74	1.78	
Halaweish	2016	Single	<3	-1.31	-2.67	0.06	
Georgoff	2017	Single	<3	-0.08	-1.32	1.16	
**Single OVERALL**				**-0.32**	**-0.89**	**0.24**	

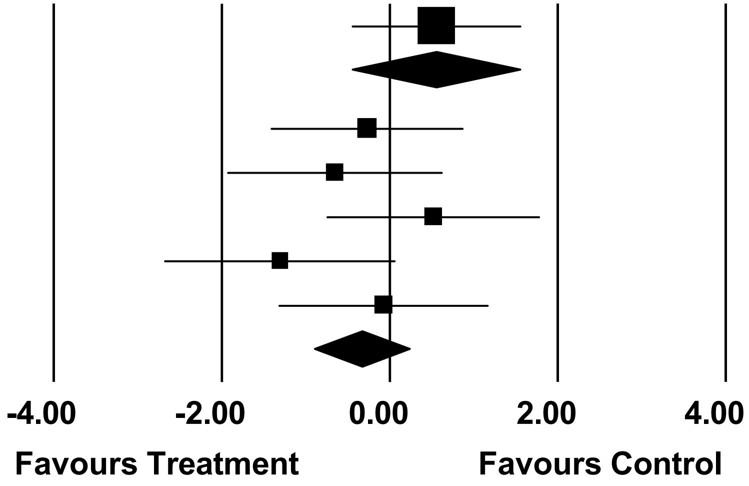

Study name	Time point	Single vs Multi	Std diff in means	Lower limit	Upper limit
Jones (i)	2016	Multi	-2.17	-2.89	-1.44
Jones (ii)	2016	Multi	-2.05	-3.06	-1.04
Jones (iii)	2016	Multi	-0.92	-1.72	-0.11
**Multi**			**-1.70**	**-2.52**	**-0.89**
Gao	2000	Single	-2.82	-4.12	-1.51
Hatori	2001	Single	-2.51	-3.91	-1.11
Lange	2006	Single	-3.80	-5.44	-2.16
Boengler	2007	Single	-2.42	-3.65	-1.19
Pagel	2007	Single	-6.92	-9.59	-4.24
Lie	2010	Single	-3.59	-4.80	-2.39
Roesner	2010	Single	-1.74	-2.89	-0.59
Smul	2011	Single	-4.26	-6.15	-2.36
Eckle	2012	Single	-12.65	-17.83	-7.46
YangZ	2013	Single	-3.36	-4.76	-1.95
**Single**			**-3.53**	**-4.45**	**-2.60**

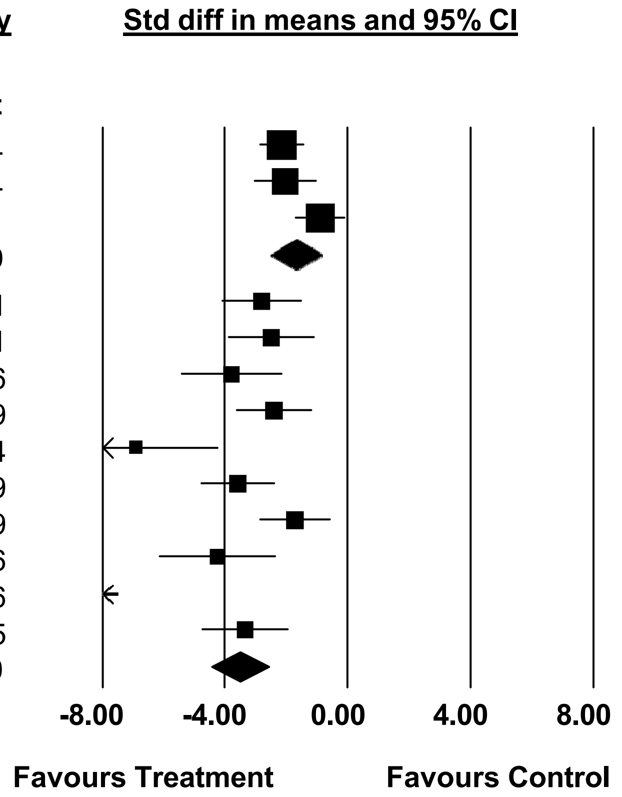

Study name	Time point	Single vs Multi	Quality Score	Std diff in means	Lower limit	Upper limit
Jones (i)	2016	Multi	≥ 3	-2.17	-2.89	-1.44
Jones (ii)	2016	Multi	≥ 3	-2.05	-3.06	-1.04
Jones (iii)	2016	Multi	≥ 3	-0.92	-1.72	-0.11
**Multi OVERALL**				**-1.70**	**-2.52**	**-0.89**
Gao	2000	Single	<3	-2.82	-4.12	-1.51
Lange	2006	Single	<3	-3.80	-5.44	-2.16
Boengler	2007	Single	<3	-2.42	-3.65	-1.19
Pagel	2007	Single	<3	-6.92	-9.59	-4.24
Lie	2010	Single	<3	-3.59	-4.80	-2.39
Roesner	2010	Single	<3	-1.74	-2.89	-0.59
Eckle	2012	Single	<3	-12.65	-17.83	-7.46
YangZ	2013	Single	<3	-3.36	-4.76	-1.95
**<3**				**-3.65**	**-4.78**	**-2.52**
Hatori	2001	Single	≥ 3	-2.51	-3.91	-1.11
Smul	2011	Single	≥ 3	-4.26	-6.15	-2.36
**≥ 3**				**-3.26**	**-4.96**	**-1.56**
**Single OVERALL**				**-3.53**	**-4.45**	**-2.60**

Std diff in means and 95% CI

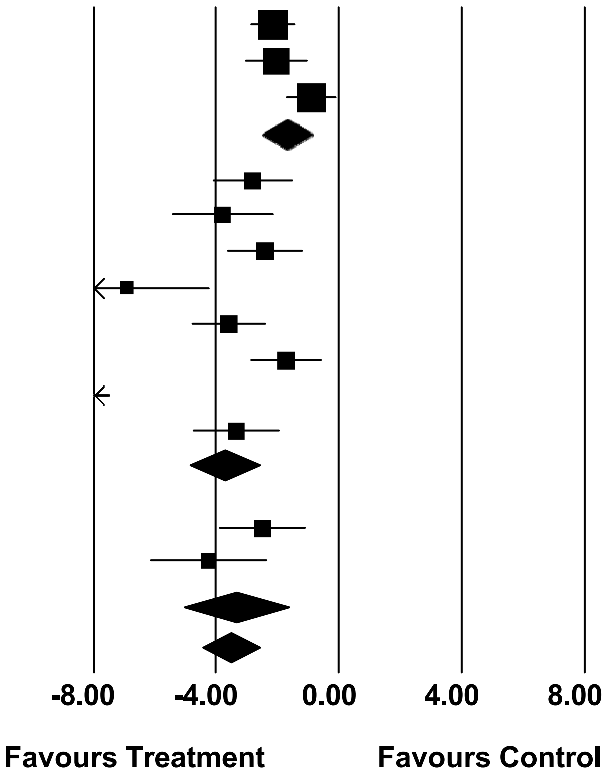

Study name	Timepoint	Single vs Multi	Std diff in means	Lower limit	Upper limit
			**Statistics for each study**		
Llovera (a)	2015	Multi	-0.03	-0.42	0.36
Llovera (b)	2015	Multi	-0.60	-1.15	-0.04
**Multi**			**-0.28**	**-0.83**	**0.27**
Becker	2001	Single	-1.12	-1.94	-0.30
Relton (a)	2001	Single	-1.28	-2.03	-0.53
Relton (b)	2001	Single	-0.63	-1.42	0.17
Liesz (a)	2011	Single	-2.35	-3.49	-1.21
Liesz (b)	2011	Single	-0.36	-1.24	0.53
Liesz (c)	2011	Single	-0.63	-1.52	0.27
Langhauser (a)	2014	Single	-0.94	-1.86	-0.02
Langhauser (b)	2014	Single	-0.09	-0.97	0.78
Neumann	2015	Single	-3.04	-4.22	-1.87
Drieu (a)	2019	Single	-0.54	-1.76	0.68
Drieu (b)	2019	Single	-0.19	-1.37	0.99
**Single**			**-0.98**	**-1.45**	**-0.51**

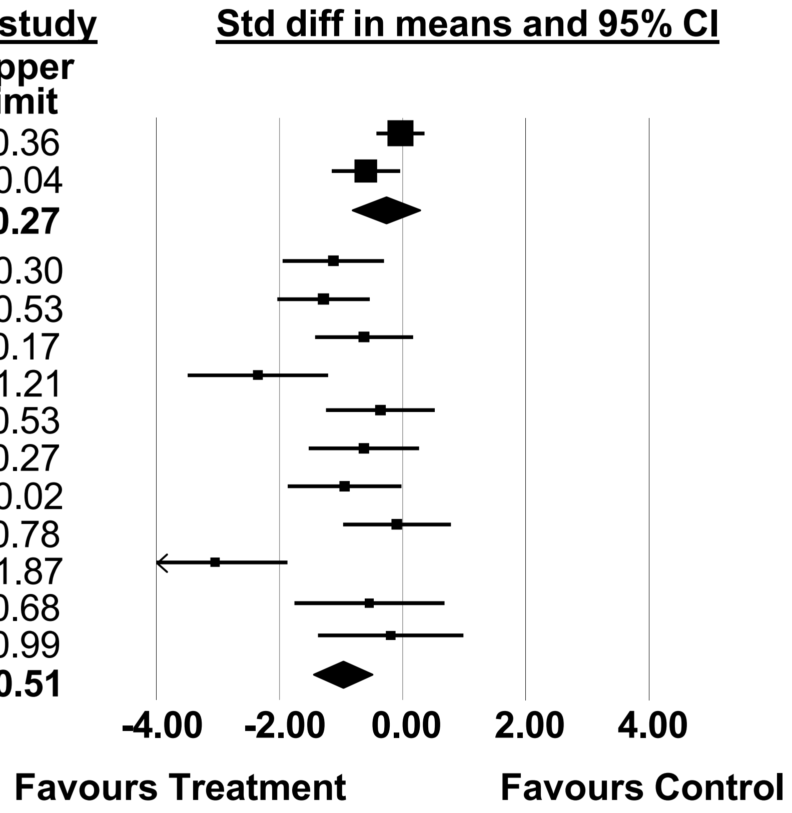

Study name	Timepoint	Single vs Multi	Quality Score	Std diff in means	Lower limit	Upper limit
Llovera (a)	2015	Multi	≥ 3	-0.03	-0.42	0.36
Llovera (b)	2015	Multi	≥ 3	-0.60	-1.15	-0.04
**Multi OVERALL**				**-0.28**	**-0.83**	**0.27**
Becker	2001	Single	<3	-1.12	-1.94	-0.30
Relton (a)	2001	Single	<3	-1.28	-2.03	-0.53
Relton (b)	2001	Single	<3	-0.63	-1.42	0.17
Liesz (a)	2011	Single	<3	-2.35	-3.49	-1.21
Liesz (b)	2011	Single	<3	-0.36	-1.24	0.53
Liesz (c)	2011	Single	<3	-0.63	-1.52	0.27
Neumann	2015	Single	<3	-3.04	-4.22	-1.87
Drieu (a)	2019	Single	<3	-0.54	-1.76	0.68
Drieu (b)	2019	Single	<3	-0.19	-1.37	0.99
**<3**				**-1.06**	**-1.60**	**-0.52**
Langhauser (a)	2014	Single	≥ 3	-0.94	-1.86	-0.02
Langhauser (b)	2014	Single	≥ 3	-0.09	-0.97	0.78
**≥ 3**				**-0.41**	**-1.42**	**0.60**
**Single OVERALL**				**-0.98**	**-1.45**	**-0.51**

Statistics for each study

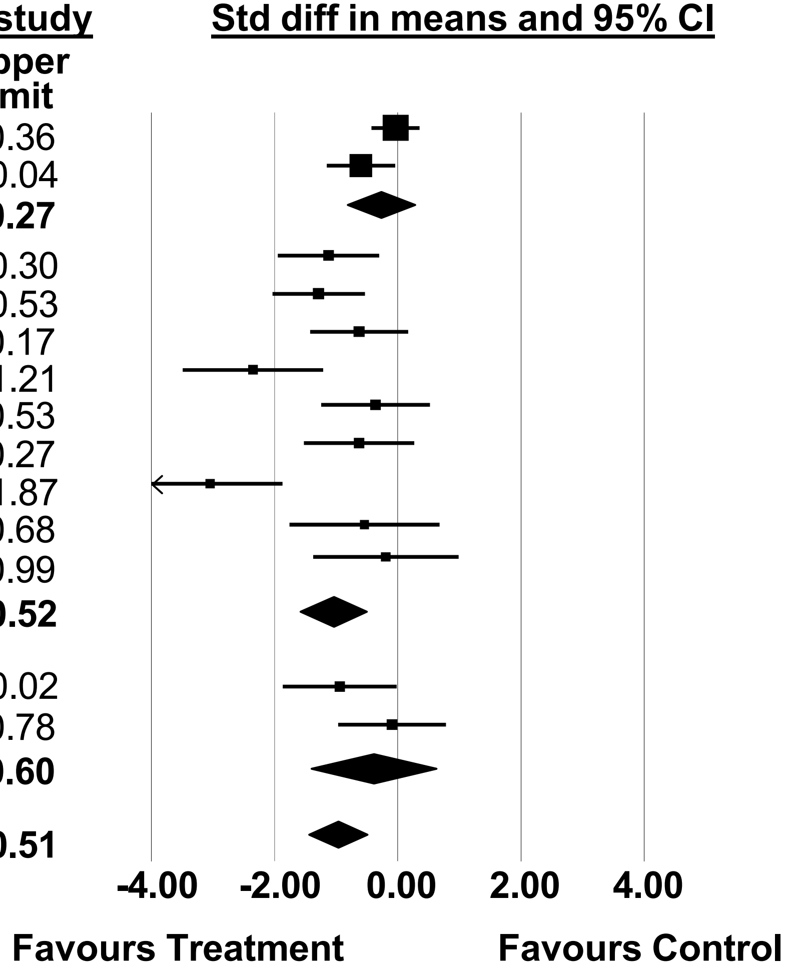

Study	Time point	Single vs. Multi	Std diff in means	Lower limit	Upper limit
Maysami	2015	Multi	-1.45	-1.90	-1.00
**Multi**			**-1.45**	**-1.90**	**-1.00**
Boutin	2001	Single	-1.04	-1.91	-0.16
Touzani	2002	Single	-1.60	-2.73	-0.48
Mulcachy (a)	2003	Single	-5.16	-7.51	-2.80
Mulcachy (b)	2003	Single	-3.82	-5.26	-2.38
Tsai	2003	Single	-2.68	-3.89	-1.48
Le Feuvre	2003	Single	-1.37	-2.54	-0.21
Craft	2006	Single	0.27	-0.69	1.23
Clark	2008	Single	-1.56	-2.75	-0.36
Pradillo (a)	2012	Single	-2.46	-3.81	-1.12
Pradillo (b)	2012	Single	-1.61	-2.92	-0.31
Denes	2014	Single	-0.74	-1.86	0.39
Girard	2014	Single	-0.64	-1.72	0.43
**Single**			**-1.69**	**-2.38**	**-1.01**

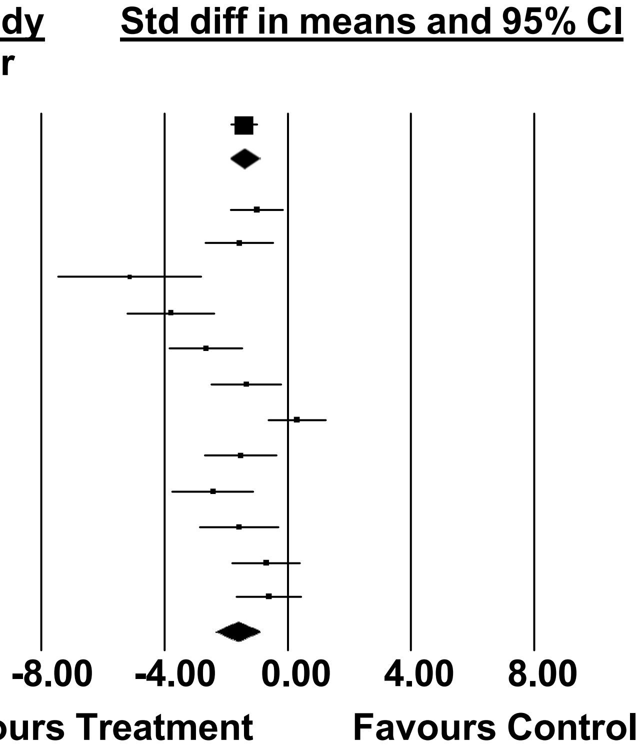

Study	Time point	Single vs. Multi	Quality Score	Std diff in means	Lower limit	Upper limit
Maysami	2015	Multi	≥ 3	-1.45	-1.90	-1.00
**Multi OVERALL**				**-1.45**	**-1.90**	**-1.00**
Boutin	2001	Single	<3	-1.04	-1.91	-0.16
Touzani	2002	Single	<3	-1.60	-2.73	-0.48
Mulcachy (a)	2003	Single	<3	-5.16	-7.51	-2.80
Mulcachy (b)	2003	Single	<3	-3.82	-5.26	-2.38
Tsai	2003	Single	<3	-2.68	-3.89	-1.48
Le Feuvre	2003	Single	<3	-1.37	-2.54	-0.21
Craft	2006	Single	<3	0.27	-0.69	1.23
Clark	2008	Single	<3	-1.56	-2.75	-0.36
Denes	2014	Single	<3	-0.74	-1.86	0.39
Girard	2014	Single	<3	-0.64	-1.72	0.43
**<3**				**-1.64**	**-2.43**	**-0.85**
Pradillo (a)	2012	Single	≥ 3	-2.46	-3.81	-1.12
Pradillo (b)	2012	Single	≥ 3	-1.61	-2.92	-0.31
≥ **3**				**-2.03**	**-2.96**	**-1.09**
**Single OVERALL**				**-1.69**	**-2.38**	**-1.01**

Statistics for each study

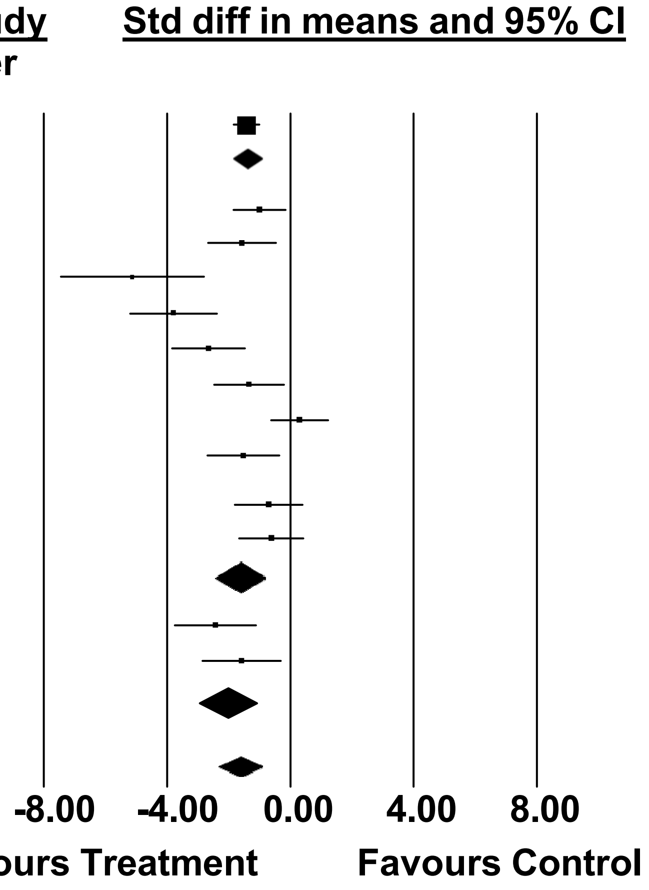

Study name	Time point	Single vs Multi	Statistics for each study		
			Std diff in means	Lower limit	Upper limit
Bramlett (i)	2016	Multi	-0.44	-1.33	0.45
Bramlett (ii)	2016	Multi	0.18	-0.62	0.98
Bramlett (iii)	2016	Multi	0.89	0.13	1.65
**Multi**			0.24	-0.51	0.98
**Single (Peng SR)**			-1.72	-2.36	-1.07

Std diff in means and 95% CI

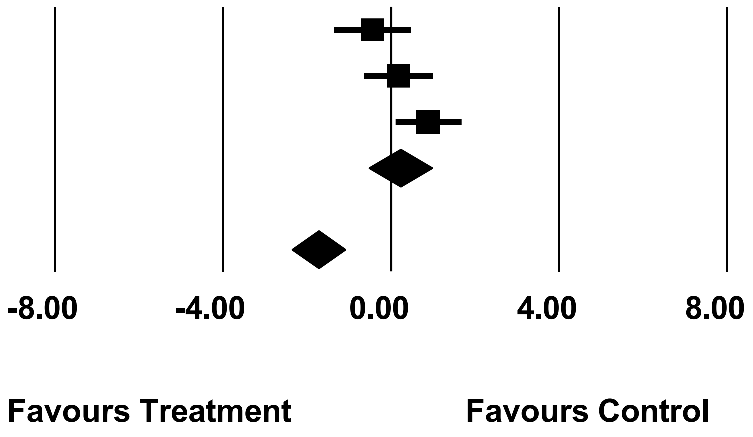

Study name	Time point	Single vs Multi	Quality Score	Std diff in means	Lower limit	Upper limit
Bramlett (i)	2016	Multi	≥ **3**	-0.44	-1.33	0.45
Bramlett (ii)	2016	Multi	≥ **3**	0.18	-0.62	0.98
Bramlett (iii)	2016	Multi	≥ **3**	0.89	0.13	1.65
**Multi OVERALL**				0.24	-0.51	0.98
**Single (Peng SR) OVERALL**			< **3**	-1.72	-2.36	-1.07

Statistics for each study

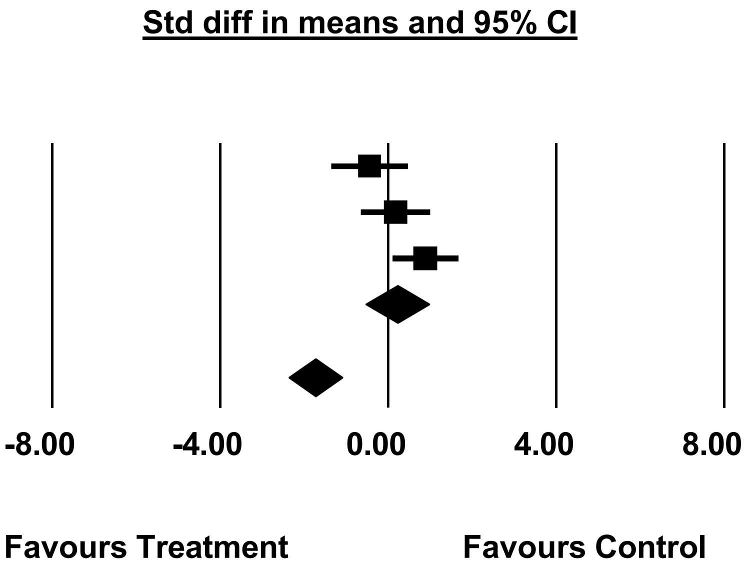

Study name	Time point	Single vs Multi	Std diff in means	Lower limit	Upper limit
			**Statistics for each study**		
Browning (FPI)	2016	Multi	0.52	-0.31	1.35
Browning (CCI)	2016	Multi	-0.60	-1.50	0.30
Browning (PBBI)	2016	Multi	-0.46	-1.29	0.37
**Multi**			**-0.17**	**-0.86**	**0.52**
Zou (i)	2013	Single	0.07	-0.73	0.87
Zou (ii)	2015	Single	-1.14	-2.00	-0.27
Caudle	2016	Single	-0.61	-1.45	0.22
Jin	2016	Single	-0.99	-2.02	0.05
**Single**			**-0.63**	**-1.18**	**-0.08**

Std diff in means and 95% CI

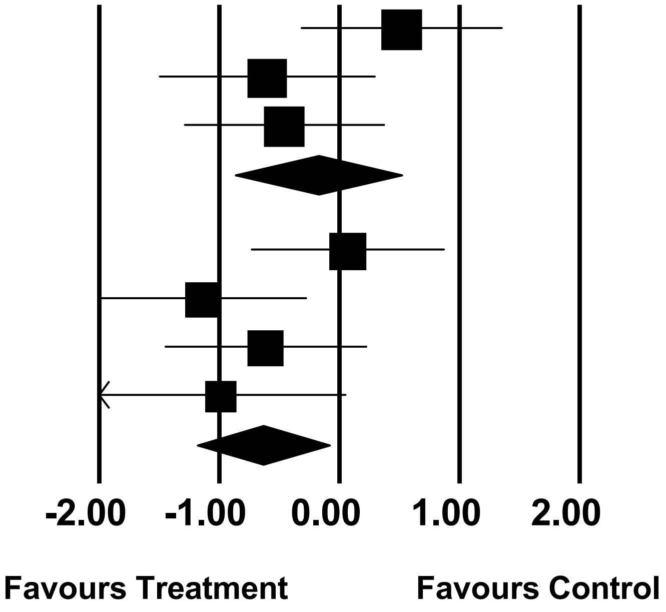

Study name	Time point	Single vs Multi	Quality Score	Statistics for each study		
				Std diff in means	Lower limit	Upper limit
Browning (FPI)	2016	Multi	≥ 3	0.52	-0.31	1.35
Browning (CCI)	2016	Multi	≥ 3	-0.60	-1.50	0.30
Browning (PBBI)	2016	Multi		-0.46	-1.29	0.37
**Multi OVERALL**				**-0.17**	**-0.86**	**0.52**
Caudle	2016	Single	<3	-0.61	-1.45	0.22
Jin	2016	Single	<3	-0.99	-2.02	0.05
**<3**				**-0.76**	**-1.41**	**-0.11**
Zou (i)	2013	Single	≥ 3	0.07	-0.73	0.87
Zou (ii)	2015	Single	≥ 3	-1.14	-2.00	-0.27
**≥ 3**				**-0.52**	**-1.71**	**0.66**
**Single OVERALL**				**-0.63**	**-1.18**	**-0.08**

Std diff in means and 95% CI

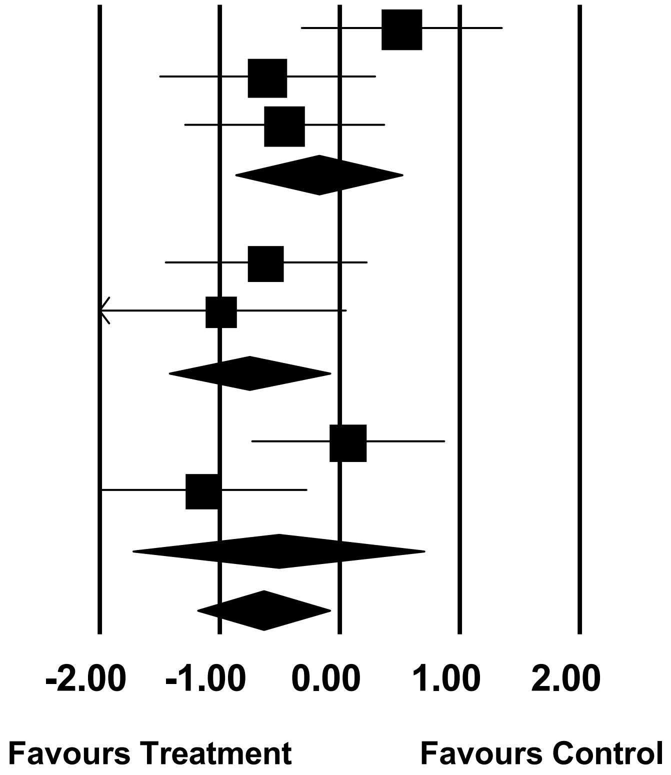

Study name	Time point	Single vs Multi	Std diff in means	Lower limit	Upper limit
Dixon (FPI)	2016	Multi	0.60	-0.28	1.47
Dixon (CCI)	2016	Multi	0.24	-0.65	1.12
Dixon (PBBI)	2016	Multi	-0.99	-1.75	-0.22
**Multi**			**-0.07**	**-1.05**	**0.91**
Scheff	1999	Single	-2.90	-4.20	-1.60
Sullivan (i)	2000	Single	-3.59	-5.59	-1.59
Fukui	2003	Single	-0.50	-1.75	0.76
Gabbita	2005	Single	1.53	0.24	2.81
Mbye	2009	Single	-2.02	-3.23	-0.82
Sullivan (ii)	2011	Single	1.52	0.41	2.63
Vlodavsky	2015	Single	-3.12	-4.58	-1.66
Karlsson	2019	Single	-1.04	-1.90	-0.19
**Single**			**-1.20**	**-2.50**	**0.09**

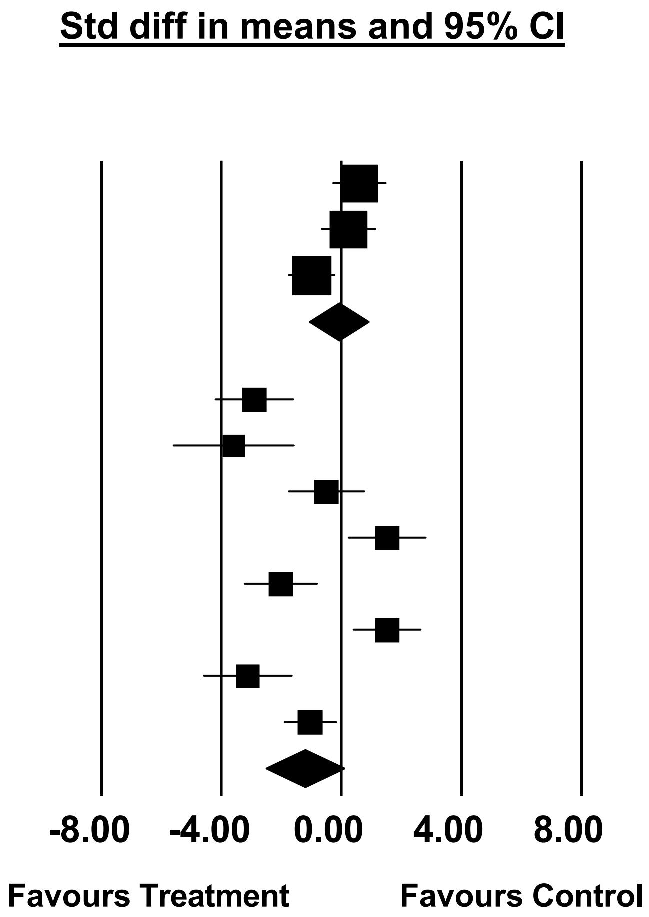

Study name	Time point	Single vs Multi	Quality Score	Statistics for each study		
				Std diff in means	Lower limit	Upper limit
Dixon (FPI)	2016	Multi	≥ 3	0.60	-0.28	1.47
Dixon (CCI)	2016	Multi	≥ 3	0.24	-0.65	1.12
Dixon (PBBI)	2016	Multi	≥ 3	-0.99	-1.75	-0.22
**Multi OVERALL**				**-0.07**	**-1.05**	**0.91**
Scheff	1999	Single	<3	-2.90	-4.20	-1.60
Sullivan (i)	2000	Single	<3	-3.59	-5.59	-1.59
Fukui	2003	Single	<3	-0.50	-1.75	0.76
Gabbita	2005	Single	<3	1.53	0.24	2.81
Mbye	2009	Single	<3	-2.02	-3.23	-0.82
Sullivan (ii)	2011	Single	<3	1.52	0.41	2.63
**<3**				-0.93	-2.63	0.77
Vlodavsky	2015	Single	≥ 3	-3.12	-4.58	-1.66
Karlsson	2019	Single	≥ 3	-1.04	-1.90	-0.19
≥ **3**				-1.99	-4.02	0.03
**Single OVERALL**				**-1.20**	**-2.50**	**0.09**

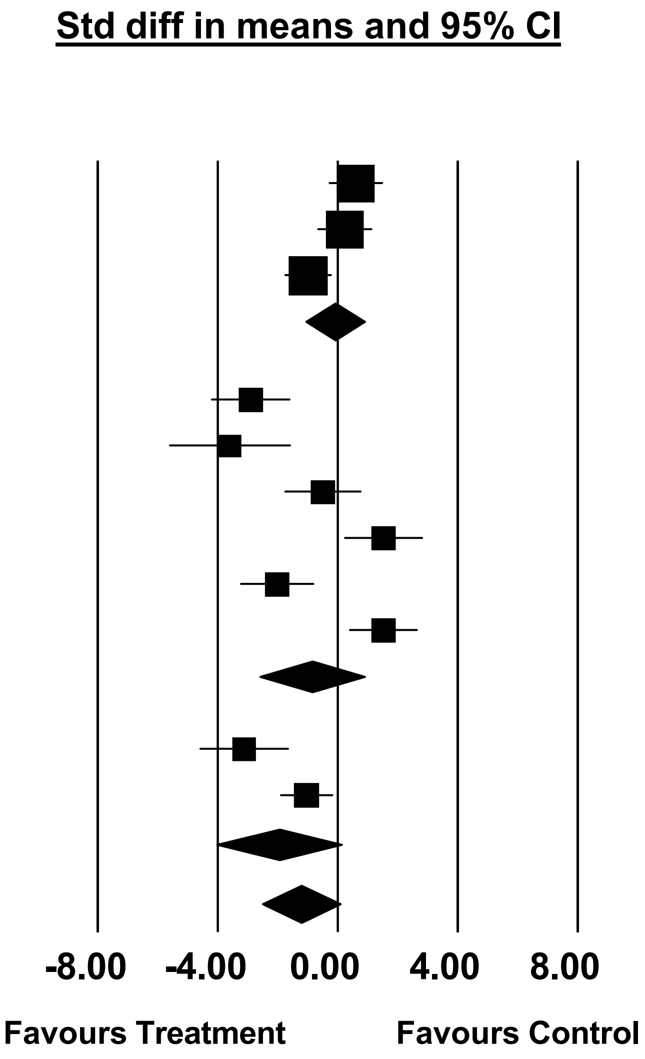

Study name	Time point	Single vs Multi	Statistics for each study		
			Std diff in means	Lower limit	Upper limit
Gill	2016	Multi	-0.13	-0.52	0.27
**Multi**			**-0.13**	**-0.52**	**0.27**
Ablamunitis	2012	Single	-0.72	-1.30	-0.14
Besançon	2018	Single	-0.72	-1.38	-0.07
**Single**			**-0.72**	**-1.16**	**-0.29**

Std diff in means and 95% CI

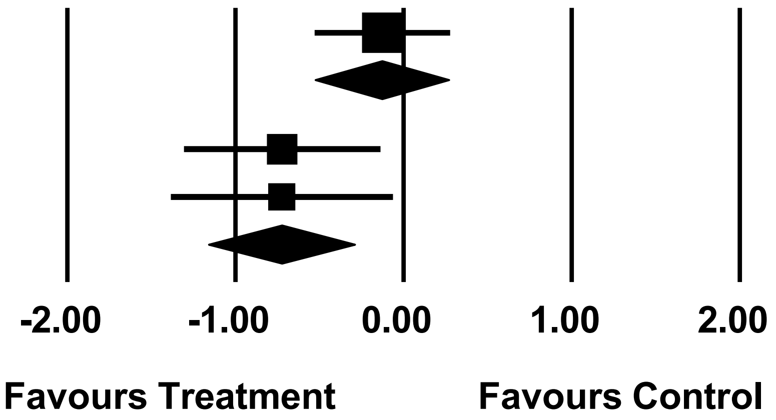

Study name	Time point	Single vs Multi	Quality Score	Statistics for each study		
				Std diff in means	Lower limit	Upper limit
Gill	2016	Multi	<3	-0.13	-0.52	0.27
**Multi OVERALL**				**-0.13**	**-0.52**	**0.27**
Ablamunitis	2012	Single	<3	-0.72	-1.30	-0.14
Besançon	2018	Single	<3	-0.72	-1.38	-0.07
**Single OVERALL**				**-0.72**	**-1.16**	**-0.29**

Std diff in means and 95% CI

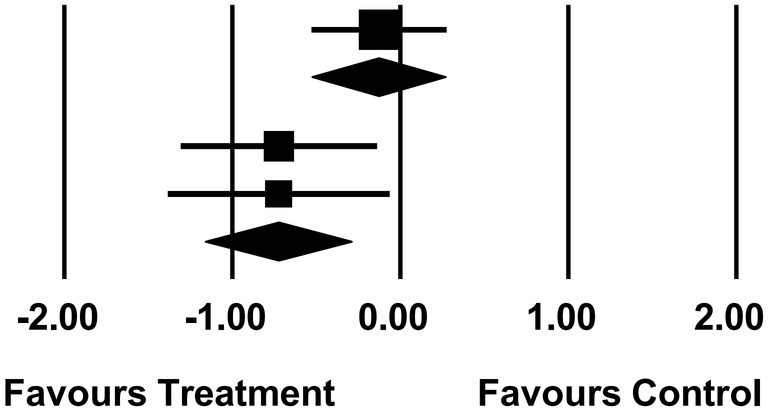

Study name	Time point	Single vs Multi	Statistics for each study		
			Std diff in means	Lower limit	Upper limit
Mountney (FPI)	2016	Multi	0.26	-0.64	1.17
Mountney (CCI)	2016	Multi	0.10	-0.83	1.03
Mountney (PBBI)	2016	Multi	0.18	-0.59	0.95
**Multi**			**0.18**	**-0.31**	**0.68**
Lu	2007	Single	0.67	-0.23	1.57
Wang	2007	Single	-0.49	-1.16	0.18
Wu	2008	Single	0.55	-0.45	1.55
Abrahamson	2009	Single	1.19	-0.15	2.54
Chauhan (i)	2010	Single	-0.28	-1.27	0.70
Chauhan (ii)	2011	Single	-0.87	-1.89	0.16
Indraswari	2012	Single	-0.43	-1.24	0.38
Vonder Haar (i)	2014	Single	-0.43	-1.46	0.60
Mountney	2016	Single	-0.45	-1.44	0.54
**Single**			**-0.13**	**-0.53**	**0.27**

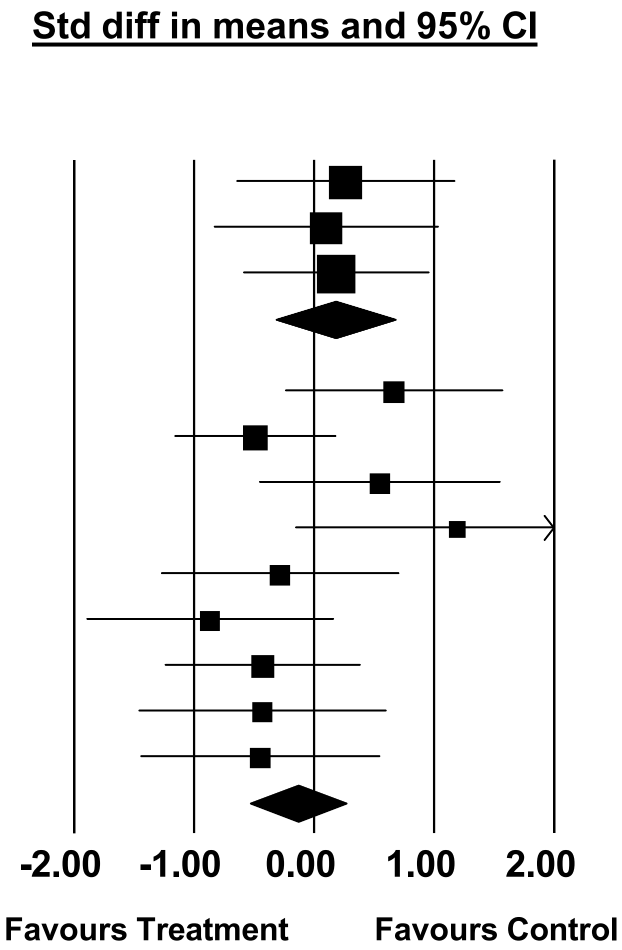

Study name	Time point	Single vs Multi	Quality Score	Std diff in means	Lower limit	Upper limit
Mountney (FPI)	2016	Multi	≥ 3	0.26	-0.64	1.17
Mountney (CCI)	2016	Multi	≥ 3	0.10	-0.83	1.03
Mountney (PBBI)	2016	Multi	≥ 3	0.18	-0.59	0.95
**Multi OVERALL**				**0.18**	**-0.31**	**0.68**
Lu	2007	Single	<3	0.67	-0.23	1.57
Wang	2007	Single	<3	-0.49	-1.16	0.18
Wu	2008	Single	<3	0.55	-0.45	1.55
Abrahamson	2009	Single	<3	1.19	-0.15	2.54
Chauhan (i)	2010	Single	<3	-0.28	-1.27	0.70
Chauhan (ii)	2011	Single	<3	-0.87	-1.89	0.16
Mountney	2016	Single	<3	-0.45	-1.44	0.54
**<3**				**-0.02**	**-0.53**	**0.49**
Indraswari	2012	Single	≥ 3	-0.43	-1.24	0.38
Vonder Haar (i)	2014	Single	≥ 3	-0.43	-1.46	0.60
**≥ 3**				**-0.43**	**-1.06**	**0.21**
**Single OVERALL**				**-0.13**	**-0.53**	**0.27**

Std diff in means and 95% CI

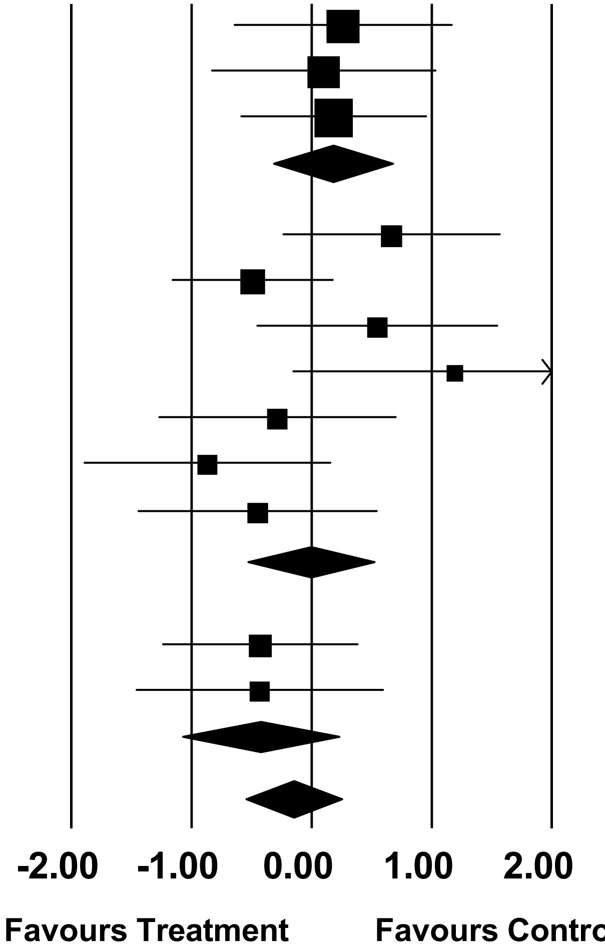

Study name	Time point	Single vs Multi	Std diff in means	Lower limit	Upper limit
			**Statistics for each study**		
Shear (FPI)	2016	Multi	-0.36	-1.24	0.52
Shear (CCI)	2016	Multi	-0.73	-1.54	0.08
Shear (PBBI)	2016	Multi	-0.25	-0.97	0.47
**Multi**			**-0.43**	**-0.89**	**0.03**
Hoane (i)	2003	Single	-1.24	-2.25	-0.23
Hoane (ii)	2006	Single	0.25	-0.41	0.90
Hoane (iii)	2008	Single	-1.64	-2.77	-0.50
Quigley	2009	Single	-0.14	-1.12	0.84
Goffus	2010	Single	-0.99	-2.03	0.05
Swan	2011	Single	-0.30	-1.36	0.77
Vondar Haar (ii)	2011	Single	-1.31	-2.30	-0.32
Peterson (i)	2012	Single	-0.98	-1.96	-0.00
Vondar Haar (iii)	2014	Single	0.19	-0.71	1.10
Peterson (ii)	2015	Single	-1.01	-2.21	0.19
**Single**			**-0.65**	**-1.10**	**-0.21**

Std diff in means and 95% CI

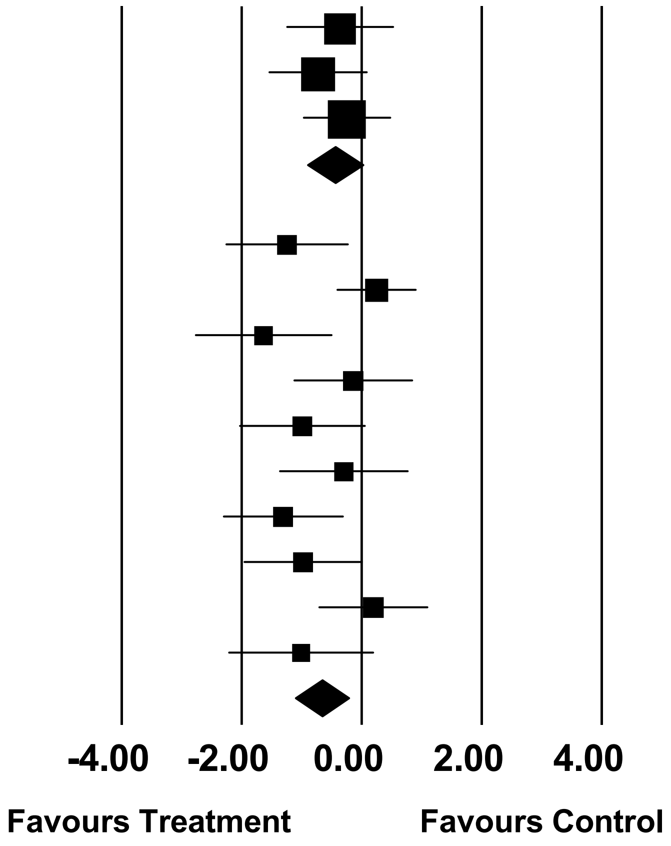

Study name	Time point	Single vs Multi	Quality Score	Std diff in means	Lower limit	Upper limit
Shear (FPI)	2016	Multi	≥ 3	-0.36	-1.24	0.52
Shear (CCI)	2016	Multi	≥ 3	-0.73	-1.54	0.08
Shear (PBBI)	2016	Multi	≥ 3	-0.25	-0.97	0.47
**Multi OVERALL**				**-0.43**	**-0.89**	**0.03**
Hoane (i)	2003	Single	<3	-1.24	-2.25	-0.23
Hoane (ii)	2006	Single	<3	0.25	-0.41	0.90
Hoane (iii)	2008	Single	<3	-1.64	-2.77	-0.50
Quigley	2009	Single	<3	-0.14	-1.12	0.84
Vondar Haar (ii)	2011	Single	<3	-1.31	-2.30	-0.32
**<3**				-0.76	-1.54	0.03
Goffus	2010	Single	≥ 3	-0.99	-2.03	0.05
Swan	2011	Single	≥ 3	-0.30	-1.36	0.77
Peterson (i)	2012	Single	≥ 3	-0.98	-1.96	-0.00
Vondar Haar (iii)	2014	Single	≥ 3	0.19	-0.71	1.10
Peterson (ii)	2015	Single	≥ 3	-1.01	-2.21	0.19
≥ **3**				-0.57	-1.07	-0.06
**Single OVERALL**				**-0.65**	**-1.10**	**-0.21**

Std diff in means and 95% CI

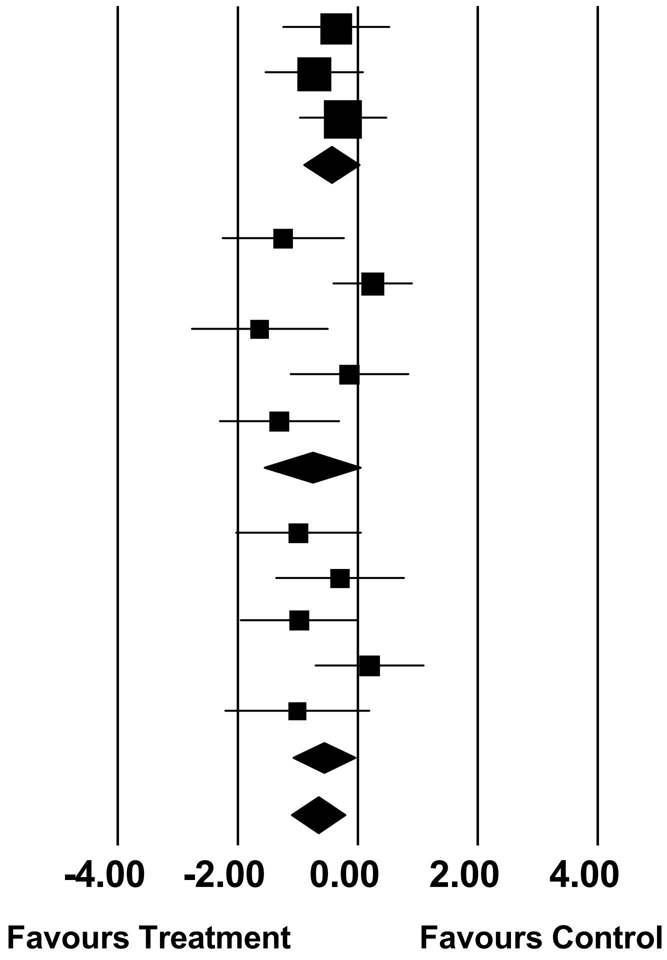

Study name	Time point	Single vs Multi	Std diff in means	Lower limit	Upper limit
Arroyo-Araujo	2019	Multi	-1.15	-1.65	-0.65
**Multi**			**-1.15**	**-1.65**	**-0.65**
Silverman (i)	2010	Single	-1.73	-2.74	-0.73
Burket	2011	Single	2.15	1.05	3.25
Mehta	2011	Single	-1.13	-1.99	-0.26
Silverman (ii)	2012	Single	-1.24	-2.15	-0.33
Aguilar-Valles	2015	Single	-2.03	-3.02	-1.04
Modi	2018	Single	-0.96	-1.89	-0.04
**Single**			**-0.84**	**-1.92**	**0.24**

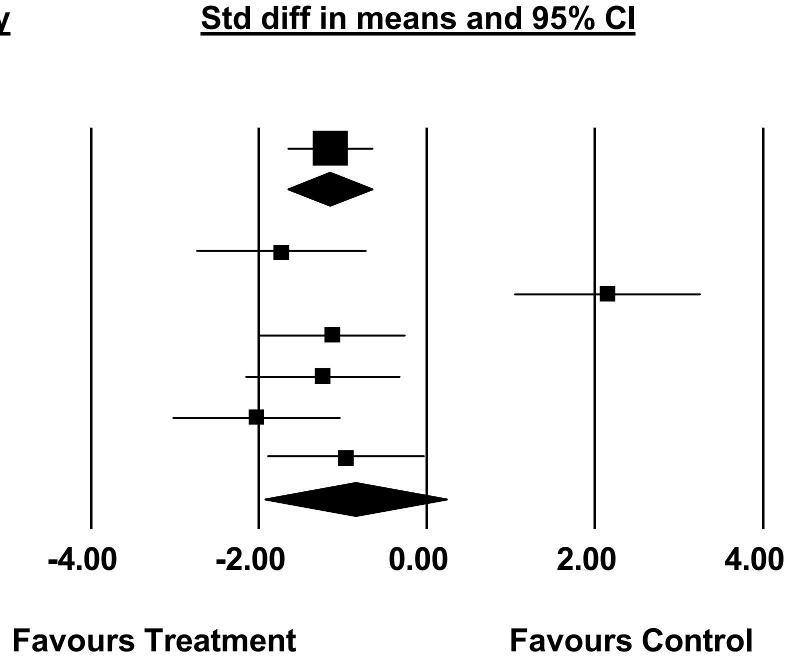

Study name	Time point	Single vs Multi	Quality Score	Std diff in means	Lower limit	Upper limit
Arroyo-Araujo	2019	Multi	≥ 3	-1.15	-1.65	-0.65
**Multi OVERALL**				**-1.15**	**-1.65**	**-0.65**
Burket	2011	Single	<3	2.15	1.05	3.25
Mehta	2011	Single	<3	-1.13	-1.99	-0.26
Silverman	2012	Single	<3	-1.24	-2.15	-0.33
Aguilar-Valles	2015	Single	<3	-2.03	-3.02	-1.04
Modi	2018	Single	<3	-0.96	-1.89	-0.04
**<3**				**-0.66**	**-1.92**	**0.60**
Silverman	2010	Single	≥ 3	-1.73	-2.74	-0.73
**≥ 3**				**-1.73**	**-2.74**	**-0.73**
**Single OVERALL**				**-0.84**	**-1.92**	**0.24**

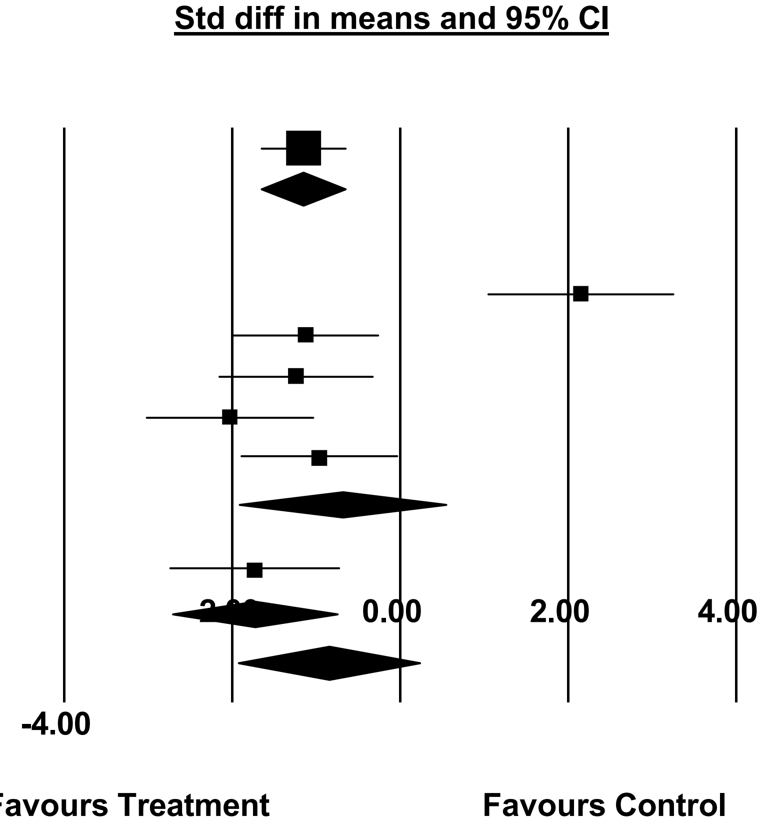

Study name	Time point	Single vs Multi	Statistics for each study: Std diff in means	Lower limit	Upper limit
Jha (CCI)	2020	Multi	-0.03	-0.75	0.69
Jha (PBBI)	2020	Multi	-1.02	-1.96	-0.09
Jha (FPI)	2020	Multi	-0.06	-0.86	0.74
**Multi**			**-0.31**	**-0.91**	**0.28**
Simard	2009	Single	-1.05	-1.98	-0.11
Zweckberger	2014	Single	-2.81	-4.04	-1.57
Xu	2017	Single	-1.35	-2.73	0.02
Jha	2019	Single	-0.11	-1.04	0.81
Gerzanich	2019	Single	-2.06	-3.21	-0.92
**Single**			**-1.42**	**-2.35**	**-0.50**

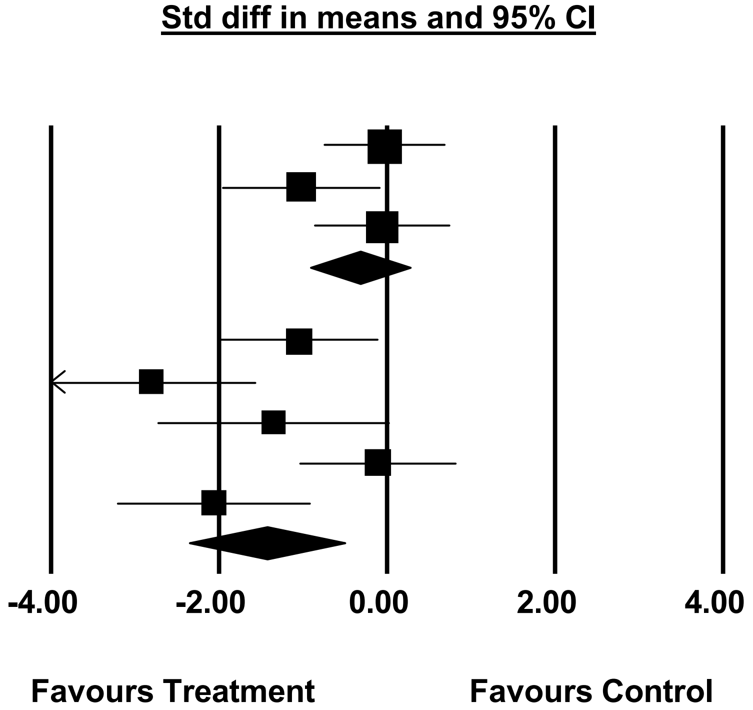

Study name	Time point	Single vs Multi	Quality Score	Std diff in means	Lower limit	Upper limit
Jha (CCI)	2020	Multi	≥ 3	-0.03	-0.75	0.69
Jha (PBBI)	2020	Multi	≥ 3	-1.02	-1.96	-0.09
Jha (FPI)	2020	Multi	≥ 3	-0.06	-0.86	0.74
**Multi OVERALL**				**-0.31**	**-0.91**	**0.28**
Simard	2009	Single	<3	-1.05	-1.98	-0.11
Xu	2017	Single	<3	-1.35	-2.73	0.02
Gerzanich	2019	Single	<3	-2.06	-3.21	-0.92
**<3**				**-1.43**	**-2.07**	**-0.79**
Zweckberger	2014	Single	≥ 3	-2.81	-4.04	-1.57
Jha	2019	Single	≥ 3	-0.11	-1.04	0.81
≥ **3**				**-1.43**	**-4.07**	**1.21**
**Single OVERALL**				**-1.42**	**-2.35**	**-0.50**

Statistics for each study

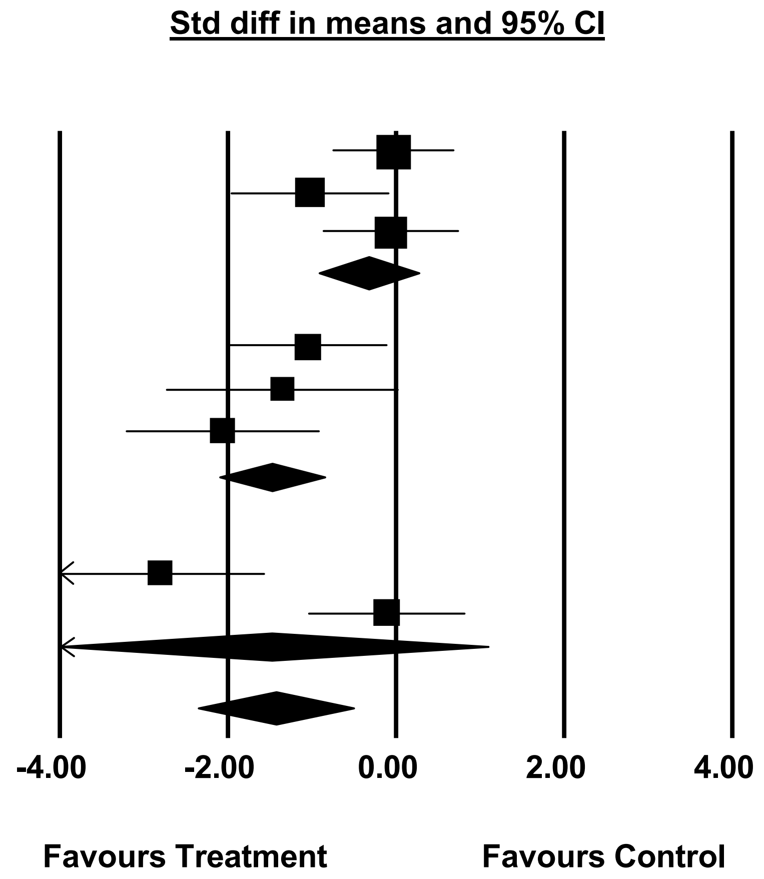

Supplement: Figure 2—source data 2. [file elife-76300-fig2-data2.pdf]
