# Supplementary material for: A systematic assessment of preclinical multilaboratory studies and a comparison to single laboratory studies
Source: eLife. 2023 Mar 9;12:e76300. doi: 10.7554/eLife.76300 (PMC10168693; doi:10.7554/eLife.76300)
Supplement: Supplementary file 1. [file elife-76300-supp1.docx]

**Supplementary file 1.** Preclinical multilaboratory search strategy.

Database: Embase Classic + Embase <1947 to 2018 January 29>, Ovid MEDLINE(R) ALL <1946 to January 29, 2018>

Search Strategy:

--------------------------------------------------------------------------------

1 Drug Evaluation, Preclinical/ (56033)

2 exp models, animal/ (1598460)

3 Animals, Laboratory/ or exp *animals, laboratory/ (99326)

4 (preclinic* or pre clinic*).ti. (35232)

5 (animal* and model*).ti. (50842)

6 or/1-5 (1750886)

7 multicenter study.pt. (227376)

8 ((cross or across) adj2 (lab or labs or laborator*)).tw. (3876)

9 ((collabor* or cooperativ* or multisite or multi-site or global) adj2 (stud* or trial* or experiment*)).tw. (53092)

10 ((multicent* or multi cent*) and (trial* or stud* or experiment*)).tw, kw. (332582)

11 or/7-10 (534463)

12 6 and 11 (3120)

13 ((multicent* or multi cent*) and (preclinic* or pre clinic* or experiment*)).ti. (162)

14 12 or 13 (3221)

**15 14 use medall (856) Medline**

16 animal experiment/ (2159290)

17 experimental animals/ (28982)

18 animal model/ (1094579)

19 (preclinic* or pre clinic*).ti. (35232)

20 (animal* and model*).ti. (50842)

21 or/16-20 (2460485)

22 multicenter study/ (401858)

23 ((cross or across) adj2 (lab or labs or laborator*)).tw. (3876)

24 ((collabor* or cooperativ* or multisite or multi-site or global) adj2 (stud* or trial* or experiment*)).tw. (53092)

25 ((multicent* or multi cent*) adj5 (trial* or stud* or experiment*)).tw. (274592)

26 or/22-25 (583441)

27 21 and 26 (3315)

28 ((multicent* or multi cent*) and (preclinic* or pre clinic* or experiment*)).ti. (162)

29 27 or 28 (3425)

30 conference abstract.pt. (2873775)

31 29 not 30 (2061)

**32 31 use emczd (1799) Embase**

33 15 or 32 (2655)

34 remove duplicates from 33 (2332)

**35 34 use medall (854) Medline**

**36 34 use emczd (1478) Embase**
