# Supplementary material for: A systematic assessment of preclinical multilaboratory studies and a comparison to single laboratory studies
Source: eLife. 2023 Mar 9;12:e76300. doi: 10.7554/eLife.76300 (PMC10168693; doi:10.7554/eLife.76300)
Supplement: Supplementary file 2. [file elife-76300-supp2.docx]

**Supplementary file 2.** PRESS review of search strategy.

***PRESS Guideline* — Search Submission & Peer Review Assessment**

**SEARCH SUBMISSION: THIS SECTION TO BE FILLED IN BY THE SEARCHER**

| Searcher’s Name: Risa Shorr | Email: rshorr@toh.ca |
| --- | --- |
| Date Submitted: 2018-01-172018-01-17 | Date Needed By: Click or tap to enter a date. |

**Systematic Review Title**

Preclinical Multilaboratory Studies

**This search strategy is: (Highlight the appropriate response)**

1. My PRIMARY (core) database strategy — First time submitting a strategy for search question and database.
2. My PRIMARY (core) strategy — Follow-up review NOT the first time submitting a strategy for search question and database. If this is a response to peer review, itemize the changes made to the review suggestions.
3. SECONDARY search strategy— First time submitting a strategy for search question and database.
4. SECONDARY search strategy – NOT the first time submitting a strategy for search question and database. If this is a response to peer review, itemize the changes made to the review suggestions.

**Database (ie, Medline, Cinahl**

Medline

**Interface (Ovid, Ebsco)**

Ovid

**Research Question. Describe the purpose of the search.**

To summarise the literature on preclinical multicenter studies

**PICO Format (Outline the PICOs for your question – ie. Patient, Intervention, Comparison, Outcome and Study Design – as applicable)**

| P | Preclinical studies |
| --- | --- |
| I | Multicenter |
| C |  |
| O |  |

**Inclusion Criteria (List criteria such as age groups, study designs, etc, to be included) [optional]**

**Exclusion Criteria (List criteria such as study designs, date limits, etc., to be excluded) *[optional]***

**Was a search filter applied?**

No

**Other notes or comments you feel would be useful for the peer reviewer?** [optional]

Here are some target articles. All are captured with the search strategy.

Searching all animal groups and study type is too huge (~10000 refs in Medline) and then not all studies are indexed as multicenter.

1**. A cross-laboratory preclinical study on the effectiveness of interleukin-1 receptor antagonist in stroke**.

Maysami S; Wong R; Pradillo JM; Denes A; Dhungana H; Malm T; Koistinaho J; Orset C; Rahman M; Rubio M; Schwaninger M; Vivien D; Bath PM; Rothwell NJ; Allan SM.

Journal of Cerebral Blood Flow & Metabolism. 36(3):596-605, 2016 Mar.

[Journal Article. Research Support, Non-U.S. Gov't]

UI: 26661169

2. **Results of a preclinical randomized controlled multicenter trial (pRCT): Anti-CD49d treatment for acute brain ischemia.**

Llovera G; Hofmann K; Roth S; Salas-Perdomo A; Ferrer-Ferrer M; Perego C; Zanier ER; Mamrak U; Rex A; Party H; Agin V; Fauchon C; Orset C; Haelewyn B; De Simoni MG; Dirnagl U; Grittner U; Planas AM; Plesnila N; Vivien D; Liesz A.

Science Translational Medicine. 7(299):299ra121, 2015 Aug 05.

[Journal Article. Multicenter Study. Research Support, Non-U.S. Gov't]

UI: 26246166

3. **The NHLBI-sponsored Consortium for preclinicAl assESsment of cARdioprotective therapies (CAESAR): a new paradigm for rigorous, accurate, and reproducible evaluation of putative infarct-sparing interventions in mice, rabbits, and pigs.**

Jones SP; Tang XL; Guo Y; Steenbergen C; Lefer DJ; Kukreja RC; Kong M; Li Q; Bhushan S; Zhu X; Du J; Nong Y; Stowers HL; Kondo K; Hunt GN; Goodchild TT; Orr A; Chang CC; Ockaili R; Salloum FN; Bolli R.

Circulation Research. 116(4):572-86, 2015 Feb 13.

[Journal Article. Multicenter Study. Research Support, N.I.H., Extramural]

UI: 25499773

4. **Different data from different labs: lessons from studies of gene-environment interaction. [Review] [83 refs]**

Wahlsten D; Metten P; Phillips TJ; Boehm SL 2nd; Burkhart-Kasch S; Dorow J; Doerksen S; Downing C; Fogarty J; Rodd-Henricks K; Hen R; McKinnon CS; Merrill CM; Nolte C; Schalomon M; Schlumbohm JP; Sibert JR; Wenger CD; Dudek BC; Crabbe JC.

Journal of Neurobiology. 54(1):283-311, 2003 Jan.

[Comparative Study. Journal Article. Research Support, Non-U.S. Gov't. Research Support, U.S. Gov't, Non-P.H.S. Research Support, U.S. Gov't, P.H.S. Review]

UI: 12486710

5. **Genetics of mouse behavior: interactions with laboratory environment**.

Crabbe JC; Wahlsten D; Dudek BC.

Science. 284(5420):1670-2, 1999 Jun 04.

[Comparative Study. Journal Article. Research Support, Non-U.S. Gov't. Research Support, U.S. Gov't, Non-P.H.S. Research Support, U.S. Gov't, P.H.S.]

UI: 10356397

6. **Animal models for protecting ischemic myocardium: results of the NHLBI Cooperative Study. Comparison of unconscious and conscious dog models.**

Reimer KA; Jennings RB; Cobb FR; Murdock RH; Greenfield JC Jr; Becker LC; Bulkley BH; Hutchins GM; Schwartz RP Jr; Bailey KR; et al.

Circulation Research. 56(5):651-65, 1985 May.

[Comparative Study. Journal Article. Research Support, U.S. Gov't, P.H.S.]

UI: 3838923

**Please copy and paste your search strategy here, exactly as run, including the number of hits per line. [mandatory]**

Database: Ovid MEDLINE(R) ALL <1946 to January 16, 2018>

Search Strategy:

--------------------------------------------------------------------------------

1 Drug Evaluation, Preclinical/ (49649)

2 exp models, animal/ (552406)

3 Animals, Laboratory/ or exp *animals, laboratory/ (25944)

4 (preclinic* or pre clinic*).ti. (15572)

5 or/1-4 (626909)

6 multicenter study.pt. (261962)

7 ((cross or across) adj2 (lab or labs or laborator*)).tw. (1811)

8 ((collabor* or cooperativ* or multisite or multi site or global) adj2 (stud* or trial* or experiment*)).tw. (25511)

9 ((multicent* or multi cent*) and (trial* or stud* or experiment*)).tw, kw. (143765)

10 6 or 7 or 8 or 9 (340472)

11 5 and 10 (927)

12 ((multicent* or multi cent*) and (preclinic* or pre clinic* or experiment*)).ti. (77)

13 11 or 12 (977)

**PEER REVIEW ASSESSMENT: THIS SECTION TO BE FILLED IN BY THE REVIEWER**

| Reviewer: Sascha Davis | adavis@toh.ca | Date Completed:2018-01-242018-01-24 |
| --- | --- | --- |

**Translation**

A- No Revision

**Boolean and Proximity Operators**

A- No Revision

**Subject Headings**

A- No Revision

**Text Word Searching**

A- No Revision

**Spelling, Syntax and Line Numbers**

A- No Revision

**Limits and Filters**

Choose an item.

**Overall Evaluation**

A- No Revision

**Additional Comments**: Click or tap here to enter text.

- Could you do “animal model*”. ti or is that too big? Or even “animal adj2 model*”. ti?
- I’m not sure if more wording could be added for the concept of cross-laboratory? – “different labs” or “simultaneous* adj3 laborator*) or (“two laborator* or “three laborator* or “four laborator*”)?
