# Supplementary material for: A systematic assessment of preclinical multilaboratory studies and a comparison to single laboratory studies
Source: eLife. 2023 Mar 9;12:e76300. doi: 10.7554/eLife.76300 (PMC10168693; doi:10.7554/eLife.76300)
Supplement: Supplementary file 3. [file elife-76300-supp3.docx]

| # | **Item Description** | Reimer, 1985 | Crabbe, 1999 | Alam, 2009 | Spoerke, 2009 | Jones, 2015 | Llovera, 2015 | Maysami, 2015 | Bramlett, 2016 | Browning, 2016 | Dixon, 2016 | Gill, 2016 | Mountney, 2016 | Shear, 2016 | Arroyo-Araujo, 2019 | Jha, 2020 | Kliewer, 2020 |
| --- | --- | --- | --- | --- | --- | --- | --- | --- | --- | --- | --- | --- | --- | --- | --- | --- | --- |
| 1 | Identification as a multicenter/multilaboratory study in title^*^ | No | No | Yes | No | Yes | Yes | Yes | No | No | No | Yes | No | No | Yes | No | No |
| 2 | Abstract states number of participating centers | Yes | Yes | Yes | Yes | Yes | Yes | No | No | No | No | No | No | No | Yes | No | Yes |
| 3 | Community based reporting guidelines listed | No | No | No | No | No | Yes | Yes | No | No | No | No | No | No | No | No | Yes** |
| 4 | Names of each participating center listed | Yes | Yes | Yes | Yes | Yes | Yes | Yes | Yes | Yes | Yes | Yes | Yes | Yes | Yes | Yes | Yes |
| 5 | List roles of participating centers (central coordinating center, experimental site) | No | Yes | Yes | Yes | Yes | Yes | Yes | Yes | Yes | Yes | No | Yes | Yes | Yes | Yes | Yes |
| 6 | No changes, or major changes to study protocol after commencement are documented | Yes | Yes | Yes | Yes | Yes | Yes | Yes | Yes | Yes | Yes | Yes | Yes | Yes | No | Yes | Yes |
| 7 | Results substantiated by repetition under a range of conditions at each site | Yes | Yes | Yes | Yes | Yes | Yes | Yes | Yes | Yes | Yes | Yes | Yes | Yes | Yes | Yes | Yes |
| 8 | Number of subjects per outcome | Yes | Yes | Yes | Yes | Yes | Yes | Yes | Yes | Yes | Yes | Yes | Yes | Yes | Yes | Yes | Yes |
| 9 | Number of measurements per subject for one experimental outcome stated | Yes | Yes | Yes | Yes | Yes | Yes | No | Yes | Yes | Yes | No | Yes | Yes | No | Yes | No |
| 10 | Number of subjects per lab | Yes | Yes | Yes | No | Yes | Yes | No | Yes | Yes | Yes | No | Yes | Yes | Yes | Yes | Yes |
| 11 | List of the total number of subjects used in each experimental group | Yes | Yes | Yes | Yes | Yes | Yes | No | Yes | Yes | Yes | No | Yes | Yes | Yes | Yes | No |
| 12 | List of all statistical tests used | Yes | Yes | Yes | Yes | Yes | Yes | Yes | Yes | Yes | Yes | Yes | Yes | Yes | Yes | Yes | Yes |
| 13 | Definition of the measure of central tendency | Yes | Yes | Yes | Yes | Yes | Yes | Yes | Yes | Yes | Yes | Yes | Yes | Yes | Yes | Yes | Yes |
| 14 | Definition of the measure of dispersion | Yes | Yes | Yes | Yes | Yes | Yes | Yes | Yes | Yes | Yes | Yes | Yes | Yes | Yes | Yes | Yes |
| 15 | Random group assignment reported | Yes | Yes | Yes | Yes | Yes | Yes | Yes | Yes | Yes | Yes | Yes | Yes | Yes | Yes | Yes | Yes |
| 16 | Description of the method of random group assignment | No | No | No | No | Yes | Yes | Yes | No | No | No | Yes | No | No | Yes | No | No |
| 17 | Experimenters blinded to group allocation during conduct of the experiment | Yes | No | No | No | Yes | Yes | Yes | Yes | Yes | Yes | No | Yes | Yes | Yes | Yes | Yes |
| 18 | Experimenters blinded to group allocation during result assessment | Yes | No | No | No | Yes | Yes | Yes | Yes | Yes | Yes | Yes | Yes | Yes | Yes | Yes | No |
| 19 | Description of an *a priori* primary outcome | Yes | Yes | Yes | Yes | Yes | Yes | Yes | Yes | Yes | Yes | Yes | Yes | Yes | No | Yes | Yes |
| 20 | Sample size for each site computed during study design | No | Yes | No | No | Yes | Yes | No | No | No | No | Yes | No | No | No | No | Yes |
| 21 | Description of the method of sample size determination | No | Yes | No | No | Yes | Yes | No | No | No | No | Yes | No | No | No | No | Yes |
| 22 | Total number of animals used for the experiment reported | Yes | Yes | Yes | Yes | Yes | Yes | No | Yes | Yes | Yes | Yes | Yes | Yes | Yes | Yes | No |
| 23 | Description of the criteria used for the exclusion of any data or subjects | Yes | Yes | Yes | No | Yes | Yes | Yes | No | No | No | No | No | No | Yes | No | Yes |
| 24 | List losses and exclusions of animals at the end of experiment | Yes | Yes | Yes | Yes | Yes | Yes | Yes | No | No | No | No | No | No | Yes | No | No |
| 25 | All outcomes described, or description of outcomes measured but not reported in results | Yes | Yes | Yes | Yes | Yes | Yes | Yes | Yes | Yes | Yes | Yes | Yes | Yes | Yes | Yes | Yes |
| 26 | Previous or pilot/preliminary studies performed and listed | No | Yes | Yes | Yes | Yes | Yes | No | Yes | Yes | Yes | Yes | Yes | Yes | Yes | Yes | Yes |
| 27 | Results were significant, or if not, null or negative outcomes included in the results | Yes | Yes | Yes | Yes | Yes | Yes | Yes | Yes | Yes | Yes | Yes | Yes | Yes | Yes | Yes | Yes |
| 28 | Limitations of the study are documented | No | Yes | Yes | Yes | Yes | Yes | Yes | Yes | Yes | Yes | No | Yes | Yes | No | Yes | No |
| 29 | Discrepancies in results across labs expected or absent, or if not, they discussed | Yes | Yes | Yes | Yes | Yes | Yes | Yes | Yes | Yes | Yes | Yes | Yes | Yes | Yes | Yes | Yes |
| **% of reported items per paper** | | 72 | 83 | 79 | 69 | 97 | 100 | 72 | 72 | 72 | 72 | 66 | 72 | 72 | 76 | 72 | 72 |
| * Multicenter or synonym (multi-site, multiple institution) | | | | | | | | | | | | | | | | | |
| ** Declaration of transparent reporting as per journal guidelines | | | | | | | | | | | | | | | | | |

**Supplementary file 3.** Completeness of reporting of preclinical multilaboratory studies for 29 reporting items.
