# Supplementary material for: A systematic assessment of preclinical multilaboratory studies and a comparison to single laboratory studies
Source: eLife. 2023 Mar 9;12:e76300. doi: 10.7554/eLife.76300 (PMC10168693; doi:10.7554/eLife.76300)
Supplement: Supplementary file 4. [file elife-76300-supp4.docx]

**Supplementary file 4.** Quality scores, effect sizes, and effect size ratios of multilaboratory and single lab studies.

| **Study** | **Was there randomization?** | **Randomization methods** | **Blinding of personnel?** | **Blinding of outcome assessment?** | **Incomplete outcome data?** | **Total score** |
| --- | --- | --- | --- | --- | --- | --- |
| **Reimer, 1985** | 1 | 0 | 0 | 1 | 0 | **2** |
| **Crabbe, 1999** | 1 | 0 | 0 | 0 | 1 | **2** |
| **Alam, 2009** | 1 | 0 | 0 | 0 | 1 | **2** |
| **Spoerke, 2009** | 1 | 0 | 0 | 0 | 1 | **2** |
| **Jones, 2015** | 1 | 1 | 1 | 1 | 1 | **5** |
| **Llovera, 2015** | 1 | 1 | 1 | 1 | 1 | **5** |
| **Maysami, 2015** | 1 | 0 | 1 | 1 | 0 | **3** |
| **Bramlett, 2016** | 1 | 0 | 1 | 1 | 0 | **3** |
| **Browning, 2016** | 1 | 0 | 1 | 1 | 0 | **3** |
| **Dixon, 2016** | 1 | 0 | 1 | 1 | 0 | **3** |
| **Gill, 2016** | 0 | 0 | 0 | 1 | 0 | **1** |
| **Mountney, 2016** | 1 | 0 | 1 | 1 | 0 | **3** |
| **Shear, 2016** | 1 | 0 | 1 | 1 | 0 | **3** |
| **Arroyo-Araujo, 2019** | 1 | 1 | 1 | 1 | 1 | **5** |
| **Jha, 2020** | 1 | 0 | 1 | 1 | 0 | **3** |
| **Kleiver, 2020** | 1 | 0 | 1 | 1 | 0 | **3** |
